# On the Conduct of the Third Stage of Labor

**Published:** 1879-06

**Authors:** DeLaskie Miller

**Affiliations:** Professor of Obstetrics and the Diseases of Children, Rush Medical College, Chicago


CHICAGO MEDICAL

JOURNAL & EXAMINER.

VOL. XXXVIII.—JUNE, 1879.—NO. 6.

[In these pages dimension and weight are expressed in terms of the Metric System, and temperature in degrees of the Centigrade Scale.]

### Original Communications.

ARTICLE I.

### ON THE CONDUCT OF THE THIRD STAGE OF LABOR. By DE LASKIE MILLER, M.D., Professor of Obstetrics and the Diseases of Children, Rush Medical College, Chicago. Read before the Chicago Gynæcological Society, April 25, 1879.

GENTLEMEN: — In venturing to occupy the time of the Society with some thoughts upon the management of the third stage of labor, I must ask the members present to descend from the elevated plane on which occult and abstruse questions are met, and where also rare and new experiences are encountered, to the common level of ordinary practice and observation. If the daily experience of the accoucheur is barren of startling incidents, it is nevertheless, to the thoughtful physician, clothed with peculiar interest: 1st, on account of the great number of cases, in ever varying forms, which constantly recur to claim his attention; and 2d, from the fact that apparently slight errors are so liable to lead to disastrous results.

Do I affirm too much in the intimation, that in a department where the recuperative forces of nature seem almost unlimited, that familiarity is liable to lead to inattention in details, which may subvert these conservative forces of nature? Let any physician of considerable experience take a retrospect of his observation — I will not say here of *his own* experience, and I feel confident he will not fail to recall instances, and possibly many which will justify the assumption.

I doubt whether a greater diversity of practice, pursued by different physicians, will anywhere else be found, than prevails in the management of the third stage of labor — in the kind of assistance offered, in the time at which it is proferred, and in the manner of its execution. I have known physicians who, especially in sparsely settled sections of the country, seldom, if ever, make the second visit to their patients after delivery, and many times not even one visit to learn by personal examination the effects and results of the labor. I have seen a physician of many years' experience, after the delivery of his patient by the forceps, introduce his hand into the uterus and forcibly deliver the placenta within ten minutes, and bid his patient good-day in a few minutes after, without making any examination to ascertain the quality of the pulse or the condition of the uterus. Instances like these might be enumerated, till gentlemen would become weary of the detail.

Believing, as I do, that the third is the most important stage of labor, I have been frequently and strongly impressed with the suspicion — which suspicion has become a conviction — that the importance of this stage of labor is too generally underestimated.

Though the accoucheur should consult his watch and govern himself by the stereotyped admonition to remain with his patient at least an hour after the delivery of the placenta, he does not thereby discharge his whole duty in all cases. The third stage of labor is not completed when the placenta is delivered, nor until certain important conditions have been secured, which ensure the safety of the patient, though it require twelve hours to accomplish these.

As a guide to our duties, we may draw useful practical lessons from a study of the natural phenomena in cases requiring no assistance. First, however, I may be allowed to refer to the normal connection between the uterus and placenta. This attachment is not effected by muscular, fibrous or cellular tissue, and does not extend deeper than the lining membrane of the uterus. The mucous membrane of the uterus lies in contact with the muscular tissue. In this, it differs from the arrangement found elsewhere, viz., that the mucous membrane is attached to organs through the intervention of a layer of sub-mucous cellular tissue. The endo-metrium receives innumerable vessels (some of which have but a single coat), which are distributed to and surround the utricular glands. This mucous membrane contributes to the development of the placenta. In this development the vessels become greatly enlarged, but their walls are not proportionally strengthened, so that the placenta is only held firmly *in situ*, while the normal conditions of gestation continue. These essential conditions are: 1st, the size of the placenta must increase *pari passu* with the segment of the uterus to which it is attached; and 2d, the counter pressure afforded by the liquor amnii must furnish the requisite amount of support to the placenta from within. Now, what is nature's mode of completing the third stage of labor? After the expulsion of the child, the uterus remains quiescent for a time, perhaps ten or fifteen minutes, then a slight dilatation of the organ is appreciable, which is immediately followed by contraction, and these alternate contractions continue till the placenta is separated and expelled from the uterus, requiring from half an hour to an hour; its extrusion from the vagina by natural forces may follow immediately or may be delayed.

These three stages in the expulsion of the placenta, viz.: 1st, the detachment; 2d, the expulsion from the uterus; and 3d, the expulsion from the vagina, are sufficiently distinct to be readily appreciated.

It is not only interesting but important to comprehend the conservative phenomena which take place in the uterus during this process of placental expulsion. Should the placenta be separated to a greater or less extent, as the child is expelled, which is usual, the contracted state of the uterus prevents hæmorrhage, and the quiescent condition permits coagula to form and seal the orifices of the sinuses, and this is repeated till the entire separation is effected. By these means the patient is protected from hæmorrage after labor.

The first deduction from this study is that *time* is an important factor in the third stage of labor—time between uterine contractions, when the organ remains quiescent to permit the small coagula to form; 2d, the impropriety of agitating the uterus by immediate compression and kneading to hasten the delivery of the placenta. In many cases, nature may be aided in completing this stage of labor, but the assistance offered should not subvert the phenomena above described. When the placenta has been expelled from the uterus, nothing is to be gained by delay; then it may be lifted out of the vagina; or again, after detachment, should it lodge centrally over the os uteri, one border may be brought down by hooking the finger over it, when it will be delivered with facility.

In difficult labors the uterus may not contract upon the placenta as promptly or as efficiently as in the case described. Then more time should be allowed. The uterine contractions should accomplish the first stage of placental delivery, viz., its detachment. Hasty forcible detachment would manifestly thwart the natural process. When a physician frequently encounters hæmorrhage in this stage of labor, the inference cannot be avoided that the error of too great haste in extracting the placenta may explain the complication.

This brings me to the enumeration of the consequences of too sudden and forcible delivery of the placenta: 1st, liability to post partum hæmorrhage, which may immediately endanger the life of the patient, or if less severe, may lead to a slow recovery; but hæmorrhage is not the only or the greatest evil to be apprehended, for this can generally be controlled; 2d, blood will be retained in the imperfectly contracted uterus and sinuses which remain patulous, then decomposition of the retained blood, a frequent cause of after-pains, will take place, and septicæmia may follow.

Is this not the possible explanation of the cause of "milk fever," which some think is necessarily attendant upon the first active function of the mammary gland? To prove that febrile movement in the system, is not necessarily due to the secretion of milk, requires only a little observation in cases managed in accordance with the principles already stated. 3d, subinvolution of the uterus, with all its secondary consequences, is another and more permanent complication which is liable to ensue.

To obviate or diminish these possible complications, various drugs and agents have been employed, all intended to fulfill one indication, viz., to secure prompt and permanent contraction of the uterus. Among these, ergot holds the first place, and is par excellence the remedy entitled to confidence, on account of its peculiar effect upon the uterus. I incline to the opinion that the only legitimate use of ergot in obstetric practice, is in post-delivery. It is many years since I have exhibited ergot to hasten the expulsion of the child. The contraction of the uterus, under the influence of this powerful agent, is so entirely different from the normal parturient action that we may well feel surprised when injury to the child or mother does not follow its use in the second stage of labor.

The profession has come to realize these dangers to some extent, and hence the caution given, not to administer ergot too early in labor or when there are obstructions to delivery. The tonic contraction of the uterus, produced by ergot, must compress the placenta continuously, and thus interfere with its function to a corresponding degree. At the same time the continuous pressure of the presenting part of the child upon the soft tissues of the mother cannot be other than injurious. I would always resort to other equally efficient and more harmless means to expedite the second stage of labor.

We hear less of hour-glass contraction and retention of the placenta, since ergot has been exhibited with caution. This leads to a brief consideration of the management in cases of retained placenta. The causes of this complication are various, but may be classified as follows: 1st, inertia of the uterus. 2d, irregular contractions of the uterus, and, 3d, abnormal adhesion of the placenta. If inertia be the cause of retained placenta, it frequently follows, and is caused by the exhaustion of muscular power, in "laborious labor," or it may be caused by the sudden artificial delivery of the child, instead of allowing the extremities of the child to remain and stimulate the uterus to complete contraction as the extremities are expelled.

In such a case, if there be no hæmorrhage, and there will not be, unless detachment to a greater or less extent has taken place, time and restoratives are essential to the safe conclusion of the labor.

If hæmorrhage be present, indicating detachment to some extent, the complete separation of the placenta by the hand, and not by traction upon the cord, would be judicious, and at the same time and by the same means, the mechanical irritation of the uterus will ensure its contraction, which may then be kept up by ergot.

Irregular contraction of the uterus must usually be overcome by mechanical means. Should there, however, be no pressing necessity for prompt delivery, as there would be if hæmorrhage complicated the case, no haste in interfering or violence in operating would be justified. The time for operating will depend much upon the condition of the patient, and will be decided by the judgment of the practitioner in any given case. It will seldom occur that anything is to be gained by waiting longer than an hour and a half. Only one additional suggestion will be offered upon this point, and that is, the admonition not to act too rapidly. Acting continuously upon the uterus, by moderately extending force, for a sufficient length of time, will accomplish more and with safety to the tissues of the uterus, than greater violence applied suddenly. Great difficulty, and even temporary defeat, may be encountered by attempting to overcome the contraction and extract the placenta too suddenly.

Abnormal adhesion of the placenta occasionally causes its retention, not as frequently however as is supposed by some. Every placenta is adherent till the uterus contracts upon it. In the normal detachment the uterus is the active agent, while the placenta is passive. Abnormal adhesion of the placenta would appear to be a reasonable explanation of hour-glass contraction of the uterus. Encystment of the placenta may result from adhesion. In this condition the placenta may be retained in utero an indefinite length of time, without causing any marked symptoms, or becoming decomposed. I remember to have delivered a placenta three days after the birth of the child. It was globular in form from the equable pressure of the uterus, which completely surrounded it, and in its structure showed not the least appearance of decomposition. Query, would it be possible in such a case for the placenta to disappear without expulsion ? It has been affirmed that the placenta has been removed by absorption. In missed labor the soft tissues may be removed thus; a more wonderful phenomenon than would be the absorption of the placenta. The practical suggestion, in adherent placenta and hour-glass contraction, is to dilate the contracted portion of the uterus, and separate the placenta by the hand and deliver, being careful to secure general contraction of the uterus as it is relieved of the afterbirth. This operation shonld be undertaken as soon as we determine the complication. Nothing is gained by delay. I would never leave the patient till the case was completed.

### RÉSUMÉ.

1st. Contractions of the uterus after the birth of the child are essential to complete the detachment and expulsion of the placenta first, and second to compress the sinuses and thus to prevent hæmorrhage.

2d. Periods of rest during this process are important, to permit the closing of the disrupted sinuses by sealing with coagula.

3d. The early agitation of the uterus by kneading and compression would defeat the conservative forces of nature in this stage of natural labor.

4th. Withhold ergot till the placenta is detached.

5th. Deliver the placenta by bringing it down edgewise with the hand, and not by traction upon the cord.

6th. Inertia of the uterus without hæmorrhage requires time and restoratives.

7th. Inertia of the uterus with hæmorrhage, introduce the hand to deliver the placenta, and at the same time secure contraction.

8th. Irregular contraction is best overcome by moderate force, continuously applied.

9th. Abnormal adhesion requires artificial interference so soon as the diagnosis is made.

